# Genotypic characterization of gut isolates from Atlantic salmon fry reveals beneficial microbes with biotechnological potential

**DOI:** 10.1128/aem.02409-25

**Published:** 2026-02-17

**Authors:** Dave Rojas Calderón, Ronja Marlonsdotter Sandholm, Thea Samskott, Mia Tiller Mjøs, Eirik Degré Lorentsen, Åsmund Kjendseth Røhr, Ingrid Bakke, Sabina Leanti La Rosa

**Affiliations:** 1 Faculty of Chemistry, Biotechnology and Food Science, Norwegian University of Life Sciences 56625, Ås, Norway; 2 Department of Biotechnology and Food Science, The Norwegian University of Science and Technology (NTNU) 8018, Trondheim, Norway; 3 Brynsløkken AS, Vestby, Norway; Universita degli Studi di Napoli Federico II, Portici, Italy

**Keywords:** Atlantic salmon fry, *Salmo salar*, gut microbiota, bacterial isolate, carbohydrate active enzymes, vitamins, *Lactococcus raffinolactis*

## Abstract

Aquaculture is a rapidly growing industry, with Atlantic salmon being one of the most intensively farmed food-producing fish in the world. While the gut microbiota is recognized to play an important role in the health and performance of various fish, knowledge of its functional capacities in salmon is gradually expanding, facilitating a better understanding of host–microbiome interactions. Nevertheless, data on the gut microbiota of juvenile salmon remain limited. Here, we isolated and genotypically characterized 11 bacteria obtained from the gut content of Atlantic salmon fry. Whole-genome sequencing using long-read Nanopore technology and functional annotation revealed their extensive metabolic versatility, including a broad array of carbohydrate-active enzymes, proteases, lipases, and (poly)phenol-degrading enzymes potentially contributing to the metabolism of feed-derived components. Additionally, all isolates possessed genomic potential for producing beneficial metabolites, including the short-chain fatty acids acetate and propionate, as well as several B vitamins and vitamin K2. Genes encoding bacteriocins and other secondary metabolites were identified, suggesting niche competitiveness against intestinal pathogens. Among the isolates, we further show that *Lactococcus raffinolactis* ASF-5 has the potential to selectively remove indigestible oligosaccharides from plant-based feeds. Indeed, this isolate grew on raffinose, an oligosaccharide associated with anti-nutritional effects in salmon. Using AlphaFold 3 models, we show that this capability is supported by a GH36 α-galactosidase and two GH32 sucrases, which can act cooperatively in the complete hydrolysis of raffinose. Finally, *L. raffinolactis* ASF-5 had a favorable safety profile for use as a feed supplement, showing susceptibility to all tested antibiotics and lacking hemolytic activity.

**IMPORTANCE:**

Although metataxonomy studies have enhanced our knowledge of the salmon gut microbiota, the functional potential of bacteria from this ecosystem remains underexplored. Well-characterized bacterial isolates are crucial to advance the growing salmon aquaculture field, allowing insights into nutrient metabolism, informing the development of beneficial interventions, and helping in identifying enzymes to improve feed digestibility. This study highlights the functional capacity of 11 bacteria isolated from the gut of salmon fry in contributing to the metabolism of carbohydrates, proteins, lipids, fatty acids, and (poly)phenols from feed, potentially increasing nutrient availability for the host or providing beneficial metabolites, including short-chain fatty acids, vitamins, and bacteriocins. Importantly, *Lactococcus raffinolactis* ASF-5 could be used to remove indigestible sugars like raffinose from plant meals. Identifying such beneficial, host-specific bacteria opens new opportunities for developing customized feed supplements or enzymes that enhance feed digestibility, to promote the health and welfare of Atlantic salmon, and enhance the economic sustainability of aquaculture.

## INTRODUCTION

The microbial communities residing in the vertebrate gastrointestinal tract (GIT) are closely linked to a wide range of host traits and functions (1). Although extensively studied in humans and other terrestrial mammals, the gut microbiota of fish has received far less attention. Indeed, we are still far from fully understanding how microbes influence metabolic functions, contribute to feed utilization, enhance immune responses, and protect against harmful microbes and disease in teleosts, including Atlantic salmon (*Salmo salar*). With the intensification of salmon aquaculture to meet the growing demand for high-protein foods, research on the gut microbiota of this fish is now moving rapidly. However, most studies to date have focused primarily on taxonomic profiling, describing how diet influences the diversity and composition of the salmon gut microbiota, still offering limited insight into the functional potential of these microbial communities (2). Understanding how microbes populate the salmon GIT, contribute to breaking down feed components, enhance nutrient availability, and produce beneficial metabolites for its host offers exciting opportunities to optimize aquaculture production systems in more sustainable and efficient ways.

A significant number of amplicon sequencing-based investigations of the gut microbiota of Atlantic salmon have characterized differences in community composition driven by diet, environmental conditions, geographical location, and pathogen exposure (3). In pre- and post-smolt and adult salmon, the prominent microbial phyla are Pseudomonadota (formerly Proteobacteria), in addition to Bacillota (formerly Firmicutes) and Mycoplasmatota (formerly Tenericutes) (4). Prevalent genera within the phylum Pseudomonadota include *Photobacterium*, *Vibrio*, and *Aliivibrio* from the order *Enterobacterales*. Other common genera, such as *Pseudomonas*, *Burkholderia*, and *Paraburkholderia*, have also been reported, along with less abundant taxa like *Sphingomonas*, *Serratia*, *Shewanella*, *Cetobacterium*, *Lelliottia*, and members of *Actinomycetia*. The phylum Bacillota includes lactic acid bacteria (LAB), such as *Lactobacillus*, *Lactococcus*, and *Leuconostoc*, along with other genera like *Bacillus* and *Clostridium* (2, 5). In addition, members of the family *Enterococcaceae* have also been identified as part of the salmonid gut microbiota (6). Mycoplasmatota has been found to be a dominant member of the core gut microbiota of adult Atlantic salmon in the saltwater phase and is predominantly represented by putative *Mycoplasma* species (4). While believed to be more stable in non-juvenile stages, the gut microbiota of fry is more dynamic and variable, often influenced by the rearing water microbiota and characterized by high interindividual variation (7, 8). The most common families detected at this lifestage are Micrococcaceae (Micrococcales), Flavobacteriaceae (Flavobacteriales), Enterobacteriaceae (Enterobacterales), and Lactobacillales (Lactococcaceae), with Mycobacteriaceae present at low to medium abundance as common transient colonizers from the environment (7, 8).

Although sequencing studies have advanced our understanding of the salmon gut microbiota, cultivation of bacteria from this ecosystem has been sporadic, largely focusing on pathogens and some commensals that have been identified through 16S rRNA gene sequencing alone (9). This limits resolution at the species or strain level and provides little insight into the functional potential of gut bacteria at the different developmental stages of the fish. The recent resurgence of cultivation-based approaches combined with state-of-the-art whole-genome sequencing techniques has recently resulted in the generation of the “Salmon Microbial Genome Atlas”, a culture collection of 211 bacterial isolates with their complete genome sequence available from pre-smolt and adult Atlantic salmon (10). In contrast, gut isolates from salmon fry remain scarce. Facilitating access to well-characterized bacterial strains provides clear added value by enabling (i) investigations into how gut bacteria contribute to the utilization of dietary components, including proteins, lipids, and carbohydrates, and produce metabolites that support host functions and immune responses; (ii) insight into the development of defined life-stage specific, commensal-based interventions as potential probiotics; and (iii) the identification of isolates and their enzymes that can be added to fish feed to remove undigestible components and improve nutrient availability.

The growing global demand for salmon has intensified the need for sustainable feed ingredients to replace fish meals, which poses ecological and economic challenges due to its reliance on finite marine resources (11). Plant proteins have become the most widely used alternative, due to their cost-effectiveness and the negative consumer perception when using terrestrial animal by-products. However, when salmon feeds contain high amounts of plant proteins to meet essential nutrient requirements, the fish growth performance is generally inferior to that achieved with fishmeal-based diets. Indeed, many nutrients in plant-based feeds are embedded within a complex carbohydrate matrix that forms a physical barrier, limiting the access of salmon digestive proteases, thus reducing the efficiency of protein digestion and amino acid absorption (12). Nutrient assimilation requires their breakdown into absorbable units by endogenous or microbial enzymes. In salmon, endogenous enzymes alone are often insufficient, so enzymatic supplements are added to improve the digestibility of feeds rich in carbohydrates. Enzymatic pretreatment of plant-based feed ingredients has been shown to improve digestibility and fish growth by removing antinutritional factors, including antigenic proteins, indigestible oligosaccharides (stachyose and raffinose), and phytic acid, which otherwise slow digestion and nutrient absorption (12). Remarkably, feed processing strategies, such as bacterial fermentation with *Bacillus* and *Lactococcus* spp. (13), or administration of beneficial bacteria, originating from the salmon gut, with metabolic capacities aligned to the carbohydrate content of the specific plant-protein source, could simultaneously provide enzymes and metabolites that may contribute to fish nutrition while increasing resilience against pathogens and disease, thereby promoting salmon health and economic sustainability.

In this work, we have genotypically characterized a collection of bacterial isolates from the GIT of salmon fry in order to assess their ability to contribute to fish nutrition and health. Genes encoding carbohydrate-active enzymes (CAZymes), enzymes for (poly)phenols degradation, proteases, and lipases were identified, pointing to abilities to metabolize feed-derived carbohydrates, phenolics, proteins, and lipids. We report their genomic potential for the production of bacteriocins and chemical compounds that may inhibit colonization of pathogenic bacteria in the salmon GIT as well as other metabolites, including vitamins and short-chain fatty acids (SCFAs), known to confer benefits to the host. Lastly, as part of the search for salmon gut beneficial microorganisms and their enzymes that can be applied to remove indigestible oligosaccharides from plant-based feeds, one isolate, *Lactococcus raffinolactis* ASF-5, was selected for further experiments to evaluate its safety and its ability to grow on raffinose.

## MATERIALS AND METHODS

### Bacterial isolation and culture conditions

The bacterial strains were isolated from the gut of healthy Atlantic salmon fry from a commercial facility. Samples were collected from three distinct aquaculture units within the same facility and represented different ages (Table S1A). For fry under 5 g, the whole GIT was dissected, while for larger fry, hindgut contents were gently squeezed out. Irrespective of the size of the fry and samples, five gut samples were pooled in a cryotube. For each individual pooled sample, a 500 µL aliquot was mixed with 500 µL of 50% glycerol, snap-frozen on dry ice, and transported to the laboratory. The samples were then thawed and homogenized using a glass rod, followed by vortexing, during which Maximum Recovery Diluent (MRD, Millipore) was added step-wise to a total of 1 mL. The homogenates were serially diluted (1:10) in MRD and streaked on Yeast extract–Casitone–Fatty Acid agar plates (DSMZ, supplemented with 15 g/L bacteriological agar), cooked meat agar plates (Sigma-Aldrich), and mucin agar plates (1% [wt/vol] hog gastric mucin, 4.5 g/L NaCl, 4.5 g/L [NH_4_]_2_SO_4_, 0.45 g/L CaCl_2_, 0.45 g/L MgSO_4_, 2.25 g/L KH_2_PO_4_, 2.25 g/L K_2_HPO_4_, 0.5 g/L cysteine, 0.05 g/L hemin, 0.001 g/L resazurin, and 16 g/L noble agar, Sigma). The agar plates were incubated under aerobic or anaerobic conditions at room temperature and were inspected daily for 3–7 days. Single colonies were picked and resuspended in 50 μL MRD, serially diluted in MRD, and streaked again on the same agar media and under the same conditions at least twice. Pure cultures were then re-streaked onto fresh Tryptone Soy Agar (TSA, Thermo Scientific) plates two more times. Twenty-four of these isolates were taxonomically classified by amplifying the full-length 16S rRNA gene using the primers 27F (5′-AGAGTTTGATCATGGCTCA-3′) and 1492R (5′-TACGGTTACCTTGTTACGACTT-3′), followed by Sanger sequencing (Eurofins Genomics) with both primers. For the resulting sequences, regions of poor quality at the 5′- and 3′-ends were trimmed off, assembled in BioEdit, and finally BLASTed against the sequences available in GenBank.

Detailed information about the origin of the samples and cultivation conditions is provided in Table S1A.

### Sequencing and assembly of bacterial genomes

Single colonies were cultured on Tryptone Soy Broth (TSB, Thermo Scientific), and genomic DNA was extracted using the DNeasy PowerSoil Pro kit (QIAGEN) following manufacturers’ instructions. DNA concentration was measured using NanoDrop One Microvolume UV-Vis spectrophotometer (ThermoFisher Scientific) and on a Qubit 3.0 fluorometer with the Qubit dsDNA High Sensitivity assay kit (Thermo Fisher Scientific). The DNA quality was measured by gel electrophoresis on a BioRad Gel Doc EZ Imager (Bio-Rad Laboratories, Inc.). Sequencing libraries were prepared using the Native Barcoding kit SQK-NBD114.24 (Oxford Nanopore Technologies, UK), followed by Oxford Nanopore 1D Genomic DNA by ligation sequencing kit SQK-LSK109 (ONT), according to the manufacturer’s instructions. The eluted libraries were then loaded onto two ONT FLO-MIN114 R10.4 flow cells and sequenced for 48 h on a MinION device operated by the MinKNOW v.24.11.10 software. POD5 files were basecalled and demultiplexed with Dorado v7.6.8 (14), using the super-accurate model (config dna_r10.4.1_e8.2_400bps_sup@v4.3.0). Following basecalling, raw reads were quality filtered using FiltLong v0.2.1 (15) with parameters set to --min_length 3,000, --keep_percent 90, and –target_bases 500,000,000. High-quality reads were subsequently assembled using Autocycler v0.4.0 (16) with Flye v2.9.5 (17), Miniasm v0.3 (18), and Raven v1.8.3 (19) as assemblers. In addition, assemblies were also generated using Flye v2.9.5 as a standalone tool with the parameters --nano-hq and –min-overlap 1,500. Both for contigs generated either with Autocycler or Flye, an initial polishing was carried out using Medaka v1.9.1 (20) using the command medaka_consensus and default settings. The medaka-polished contigs were further refined using Racon v1.5.0 (21) with the arguments -m 8, -x 6, -g 8, and -w 500. Assembly quality was evaluated using CheckM2 v1.1.0 (22) with the parameter --allmodels. For each isolate, the assembly with the fewest contigs, lowest contamination, and highest completeness was selected between the Autocycler and Flye results. Genomes were dereplicated at the strain level (99% average nucleotide identity) using dRep v3.6.2 (23).

### Genome taxonomy and functional annotation

GTDB-Tk v2.4.0 (24) with GTDB release 220 was used to assign taxonomy for all genomes. Functional annotations were obtained with DRAM v1.5.0 (25) with the following databases: Uniref90, PFAM-A, KOfam, and dbCAN-V11 (all downloaded in August 2025). The subcellular localization of DRAM-annotated proteases and lipases was obtained using SignalP6 v6.0 (26).

To assess the genomes for the presence of genes encoding enzymes involved in vitamin B production, an in-house script was developed. The script searches for Enzyme Commission (EC) numbers corresponding to enzymes involved in vitamin B biosynthetic pathways, including those for thiamin (vitamin B1), riboflavin (vitamin B2), niacin (vitamin B3), pantothenate (vitamin B5), pyridoxine (vitamin B6), biotin (vitamin B7), folate (vitamin B9), cobalamin (vitamin B12), and menaquinone (vitamin K2), within DRAM-annotated proteins. The list of selected EC numbers was compiled from the Kyoto Encyclopedia of Genes and Genomes after a comprehensive literature search (27, 28). A genome was considered positive for a particular vitamin biosynthetic pathway if at least 75% of the genes encoding enzymes in the pathway of interest were present.

Genes or chromosomal mutations leading to antimicrobial resistance were annotated using ResFinder v4.7.2 (29, 30) with settings –species other, --min_cov 0.6, --threshold 0.8, --acquired, and –point. The databases employed for running ResFinder were downloaded with the software in July 2025. Bacteriocin predictions were obtained using Bagel4 (31). The antibiotics and Secondary Metabolites Analysis Shell (antiSMASH v8.0 [32]) was used to predict biosynthetic gene clusters involved in secondary metabolite production using relaxed detection strictness and all extra features on.

### Phylogeny

Phylogenetic relationships among the strains obtained in this study were inferred using the concatenated alignment of 120 ubiquitous single-copy proteins obtained from GTDB-Tk. The maximum-likelihood phylogenetic tree was constructed using IQ-Tree v3.0.1 (33) with settings “-m MFP -bb 1000 -nt 16.” The best amino acid substitution model (LG + F + I+G4) was automatically selected by ModelFinder (34) using the Bayesian information criterion. The tree was visualized in R v4.3.3 (35) with the packages tidyverse v2.0.0 (36), ggtree v3.10.0 (37), tidytree v0.4.6 (38), and ape v5.8.1 (39). The genome of *Prochlorococcus marinus* subsp. *marinus* str. CCMP1375 (RefSeq GCF_000007925.1) was used as the outgroup.

### Viral contig prediction

Genomes were processed with VirSorter2 v2.2.4 (40) in virome decontamination mode to identify viral nucleotide signatures of ssDNA and dsDNA viruses, employing the parameters –prep-for-dramv and –min-length 1,500. The completeness level of the predicted prophage regions was evaluated using CheckV v1.0.3 with an “end-to-end” parameter and the database v.1.5. Viral sequences were annotated using DRAM-v v1.5.0 (25) using default parameters.

### Mapping of 16S rRNA genes to publicly available amplicon datasets

The 16S rRNA amplicon datasets from Atlantic salmon gut samples were downloaded from the following NCBI BioProjects: PRJEB39298 (41), PRJNA498084 (42), PRJNA555355 (43), PRJNA590084 (44), PRJNA594310 (45), PRJNA650141 (46), PRJNA730696 (47), PRJNA733893 (48), PRJNA824235 (49), PRJNA824256 (50), PRJNA866155 (51), PRJEB60544 (2), PRJEB60545 (2), PRJNA662976 (52), and PRJNA762510 (53). FASTQ files from each BioProject were downloaded using the fasterq-dump tool v3.0.1 from the SRA Toolkit (54). Read quality was assessed, and sequencing adapters were removed using Fastp (55), with quality trimming applied at a Phred score threshold of >25. The DADA2 pipeline (56) v1.34.0 was then used for reads denoising, merging, and screening for chimeric sequences, which were subsequently removed, to finally obtain amplicon sequence variants (ASVs) for each BioProject. ASVs were compared against a custom database comprising 63 distinct 16S rRNA gene sequences extracted using Barrnap v0.9 (57) from the 11 genomes. The full set of ASVs from all amplicon datasets was queried using ncbi-blast-2.17.0 (30) with settings blastn, -outfmt 6, and -max_target_seqs 10. Blast hits were filtered for ≥97% identity (“pident”). For each genome, the best hit (if any) to each amplicon dataset was retained. Results were visualized using ggplot2 v3.4.0 (58) in R v4.3.3 (35).

### Comparative genomic analysis of *L. raffinolactis* ASF-5

For comparative genomic analysis of *L. raffinolactis* ASF-5, the genome sequences of 42 publicly available strains were downloaded from NCBI (Table S3). The phylogenetic tree was constructed using the concatenated alignment of 120 ubiquitous single-copy proteins obtained from GTDB-Tk. The phylogenetic tree was constructed using IQ-Tree v3.0.1 (33) with settings “-m MFP -bb 1,000 -nt 16.” The best amino acid substitution model (JTTDCMUT + F + R3) was automatically selected by ModelFinder (34) using the Bayesian information criterion. The tree was visualized in R v4.3.3 (35) with the packages tidyverse v2.0.0 (36), ggtree v3.10.0 (37), tidytree v0.4.6 (38), and ape v5.8.1 (39). *Pseudolactococcus plantarum* NBRC 100936 (RefSeq GCF_001591745.1) was employed as the outgroup.

### Antibiotic sensitivity test and hemolytic activity

Antibiotic susceptibility of *L. raffinolactis* ASF-5 was assessed using the agar disk diffusion method following the Clinical and Laboratory Standards Institute (CLSI) guidelines (59). A 100 μL bacterial inoculum (10⁷–10⁸ CFU/mL) was evenly spread onto TSA (Oxoid) plates. Antibiotic disks (Becton Dickinson Microbiology Systems, USA) were then applied to the surface, and plates were incubated at 20°C for 48 h. The diameters of inhibition zones were measured in millimeters. The following antibiotic disks were tested: oxacillin (1 μg), ampicillin (10 μg), tetracycline (10 μg and 30 μg), vancomycin (30 μg), rifampicin (5 μg), erythromycin (15 μg), and amoxicillin (25 μg). Disk diffusion test results were interpreted according to the breakpoints recommended by the CLSI (59). Isolates with inhibition zones ≤14 mm were classified as resistant (R), those with zones >20 mm as susceptible (S), and zones between 15 and 19 mm as intermediate. *Lactococcus garvieae* ATCC 49156 was used as a positive control based on known antibiotic resistance (60).

Hemolytic activity was assayed by culturing *L. raffinolactis* ASF-5 on blood agar plates supplemented with 7% (vol/vol) defibrinated horse blood (Sigma-Aldrich). Overnight TSB cultures of the strain tested were diluted 1:100, spotted onto fresh plates, and incubated at 30°C or 20°C for 24 h under either aerobic or anaerobic conditions. The absence of a transparent halo surrounding the colonies was interpreted as an indication of a negative hemolytic phenotype.

### Growth on raffinose

*L. raffinolactis* ASF-5 and *L. garvieae* ATCC 49156 were routinely cultured in De Man, Rogosa, and Sharpe (MRS) agar (Sigma) at 15 °C overnight. The growth experiments performed with individual carbohydrates as a sole carbon source were conducted in 200 µL cultures in 96-well microtiter plates. The bacteria were pre-grown overnight in MRS base (per liter [L], 10 g peptone, 8 g meat extract, 4 g yeast extract, 1 g Tween 80, 2 g ammonium citrate, 5 g sodium acetate, 0.2 g magnesium sulfate, 0.05 g manganese sulfate, and 2 g dipotassium phosphate) supplemented with 0.5% (vol/vol) glucose. The overnight culture (5 μL) was used to inoculate 195 μL MRS base supplemented with 0.5% (vol/vol) D-(+)-raffinose penta-hydrate (Sigma). MRS base supplemented with 0.5% (vol/vol) glucose and no carbohydrate was used as controls. Beta-glucan from barley (Megazyme) was used as a negative control, as *L. raffinolactis* ASF-*5’*s genome lacks genes encoding enzymes required to depolymerize and grow on this complex substrate. Growth was assessed by measuring the optical density (absorbance) at 600 nm (OD_600_) at 15 min intervals for 18 h by using a Varioskan LUX Multimode Microplate Reader (Tecan). All experiments were performed in triplicate.

### Predictive models of LrGH32 and LrGH36 topology

Protein structures of two glycoside hydrolase (GH) family 32 (GH32) sucrases and an α-galactosidase GH36 from *L. raffinolactis* ASF-5 were modeled individually using AlphaFold 3 (DeepMind/Isomorphic Labs; database release 15 April 2025) (61). Each enzyme was predicted in complex with raffinose as the bound substrate. For each enzyme–ligand complex, 10 independent ligand-binding poses were generated using AlphaFold 3’s ligand-cofolding workflow. The highest-scoring predicted pose (based on the model’s internal confidence metric for protein–ligand interface quality) was selected for subsequent structural and comparative analysis. All modeling was performed under default AlphaFold 3 parameters, with full multiple sequence alignment and template search enabled against the 15 April 2025 structural database release.

## RESULTS AND DISCUSSION

### General genomic features of bacterial isolates recovered from the gut of salmon fry

Microbial cultivation remains indispensable for isolating bacterial strains and provides a supply of isolates to be included in culture collections (10, 62, 63). These efforts continue to enable the linking of microbial genotypes with phenotypes, supporting experimental validation of predicted functions, eventually advancing our understanding of how gut bacteria can influence host metabolism and physiology. In addition, it has the potential to provide host-specific probiotics/synbiotics for future microbiota-targeted interventions.

**Fig 1:**
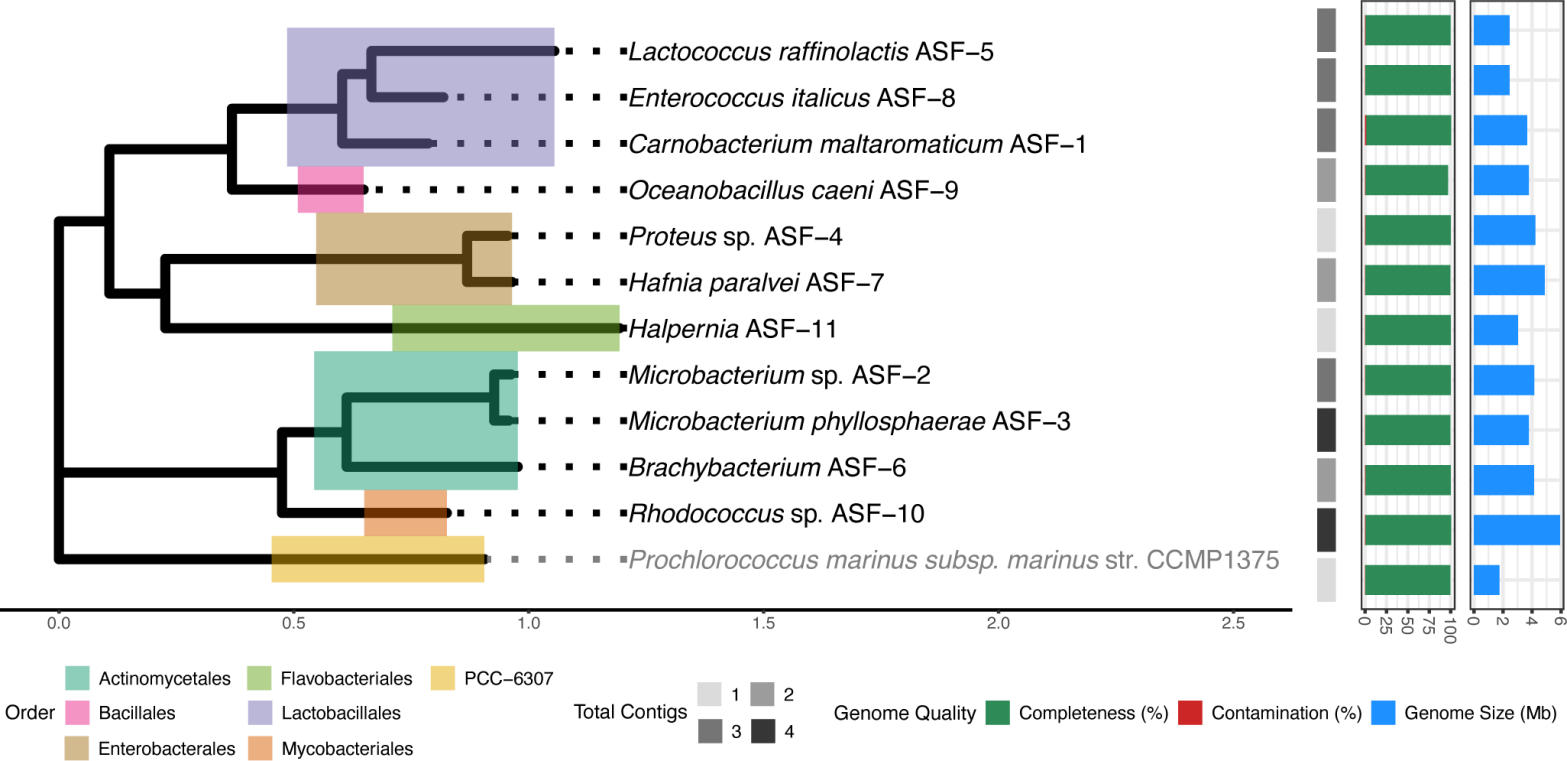
Phylogenetic tree displaying the evolutionary relationships of the 11 salmon gut bacterial isolates analyzed in this study. The genome of *P. marinus* subsp. *marinus* str. CCMP1375 (RefSeq GCF_000007925.1) was used as the outgroup. Different coloring over the tree branches depicts the genome taxonomic order. The heatmap with gray shades shows the total number of contigs per genome. The horizontal bar plots displayed next to the heatmap depict genome quality (red bars for contamination and green bars for completeness) and genome size (blue bars).

To broaden the repertoire of gut isolates derived from salmon fry, we initially obtained a total of 24 isolates from the gut contents of 20 fish. After preliminary identification based on 16S rRNA gene sequencing, we performed whole-genome sequencing using long-read Oxford Nanopore Technology. Following processing and dereplication, the final dataset consisted of 11 unique genomes (Fig. 1 and Table S1B). Genome assembly produced circular chromosomes in all cases. In nine genomes, assemblies consisted of 2–4 contigs, comprising one chromosomal and one to three plasmid sequences. Taxonomic classification using the Genome Taxonomy Database Toolkit (GTDB-tk) showed that the majority of the genomes were affiliated with the Actinomycetales (*n* = 3) and the Lactobacillales (*n* = 3) orders, followed by the Enterobacterales (*n* = 2), Bacillales (*n* = 1), Flavobacteriales (*n* = 1), and Mycobacteriales (*n* = 1). Genome sizes ranged from 3.78 to 4.1 Mb for Actinomycetales (average of 4 Mb), 2.4 to 3.6 Mb for Lactobacillales (average of 2.9 Mb), and 4.2 to 4.8 Mb for Enterobacterales (average of 4.6 Mb). *Rhodococcus* sp. ASF-10 had the biggest genome, with a size of 5.9 Mb. The genome sizes observed across the isolates fall within expected ranges for their respective taxonomic groups. Differences in genome sizes were reflected in the number of predicted genes, with the lowest number in *L. raffinolactis* ASF-5 and the highest in *Rhodococcus* sp. ASF-10, with 2,470 and 5,538 predicted genes, respectively. Notably, two isolates represent species that have not previously been reported in the genera *Halpernia* and *Brachybacterium* (Table S1B).

### Detecting the bacterial isolates in publicly available 16S rRNA amplicon sequencing studies

**Fig 2:**
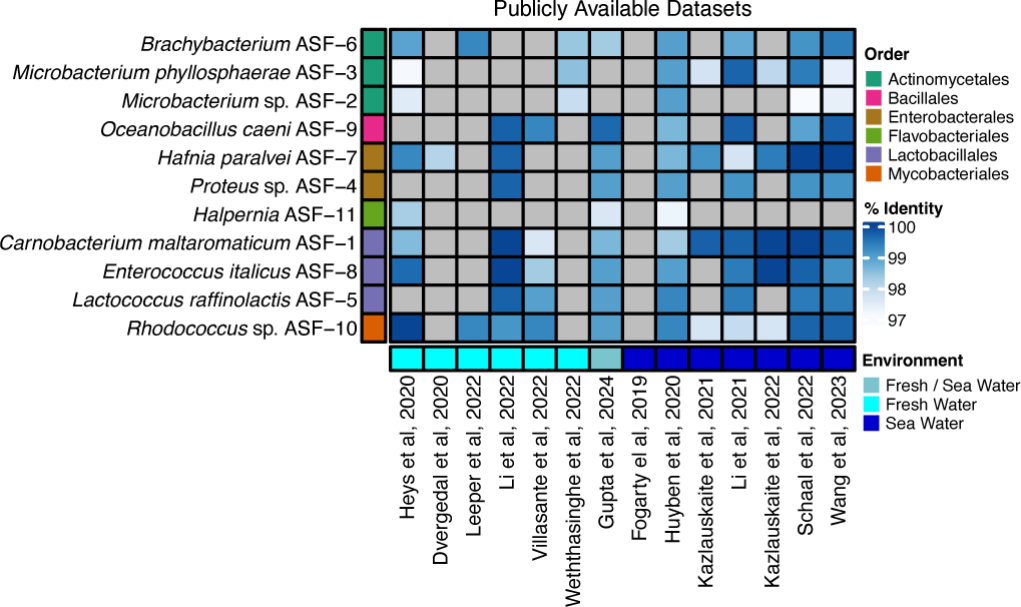
Detection of the 11 isolates from the gut of Atlantic salmon fry (y-axis) in selected publicly available 16S rRNA gene amplicon datasets (x-axis) based on alignment of 16S rRNA gene sequences. The coloring depicts the percent identity of the 16S rRNA gene alignment. At the 97% identity level to ASVs, the 11 isolates were detected in publicly available 16S rRNA gene datasets from either *in vivo* or *in vitro* trials. Gray cells represent a percent identity below 97%. Colors along the x-axis indicate the environment of the salmon sampled, while colors along the y-axis denote the taxonomic order of each isolate.

Next, we asked whether the 11 bacterial isolates could be detected in the gut microbiome of salmon across various developmental stages and lifestyles. Since the long-read DNA sequencing ensured that all genomes encoded full-length 16S rRNA genes, we leveraged this feature to probe for their presence across the extensive amplicon datasets that characterize salmon gut microbiome studies (41–53). At 97% identity cut-off, comparison of the 16S rRNA gene sequences from the 11 bacterial isolates in this study with ASVs from 14 previous studies revealed that all isolates were present in gut microbiome datasets derived from juvenile and adult salmon (Fig. 2). The 11 bacteria were detected not only in Norwegian salmon microbiomes but also in gut samples from wild and farmed Atlantic salmon in Scotland, the United Kingdom, and Chile.

### Predicted metabolic functions encoded in the genomes of the bacteria derived from the gut of salmon fry

**Fig 3:**
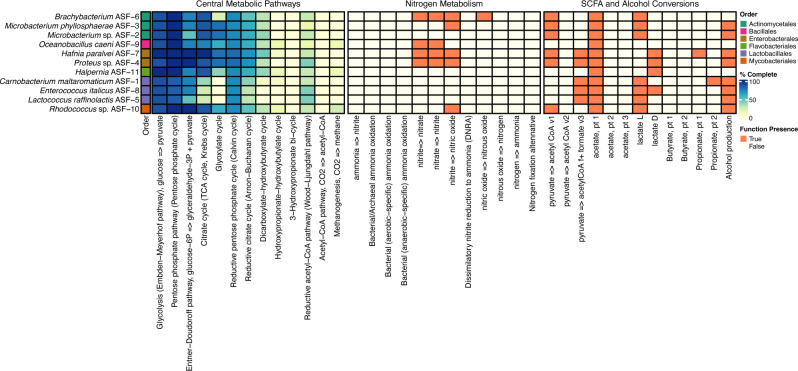
Heatmap showing the completeness (%) of central metabolic pathways and the presence of functions related to nitrogen metabolism, SCFA, and alcohol conversions (x-axis) for the 11 bacteria isolated from the gut of Atlantic salmon fry (y-axis) in this study. Pathway completeness is indicated by a color gradient from dark blue (100%) to light yellow (0%). The presence or absence of a specific gene or pathway is denoted by orange or yellow boxes, respectively. Colors in the y-axis denote the taxonomic order of each isolate.

Using functional annotation databases, we next explored the metabolic potential of each isolated bacterial strain (Fig. 3 and Table S1C). As expected, core metabolic pathways (e.g., glycolysis, pentose phosphate pathway, and tricarboxylic acid cycle) were largely similar among the 11 strains. All strains presented genes and pathways that could lead to potential beneficial metabolites in the salmon gut, such as the SCFAs acetate (all strains) and the organic acid lactate (all strains except for *Oceanobacillus caeni* ASF-9). *Carnobacterium maltaromaticum* ASF-1 and *Hafnia paralvei* ASF-7 displayed potential for propionate production. Nitrogen cycling varied across the 11 genomes, with *Brachybacterium* ASF-6, *O. caeni* ASF-9, *H. paralvei* ASF-7, and *Proteus* sp. ASF-4 are predicted to convert nitrite into nitrate, nitrate into nitrite, and nitrite into nitric oxide (with the exception of *O. caeni* ASF-9 for the latter pathway). *Brachybacterium* ASF-6 had the genomic capacity to convert nitric oxide into nitrous oxide, while *Rhodococcus* sp. ASF-10 and *Microbacterium phyllosphaera*e ASF-3 also displayed a predicted capacity to convert nitrite into nitric oxide.

#### Potential for dietary carbohydrate utilization

**Fig 4:**
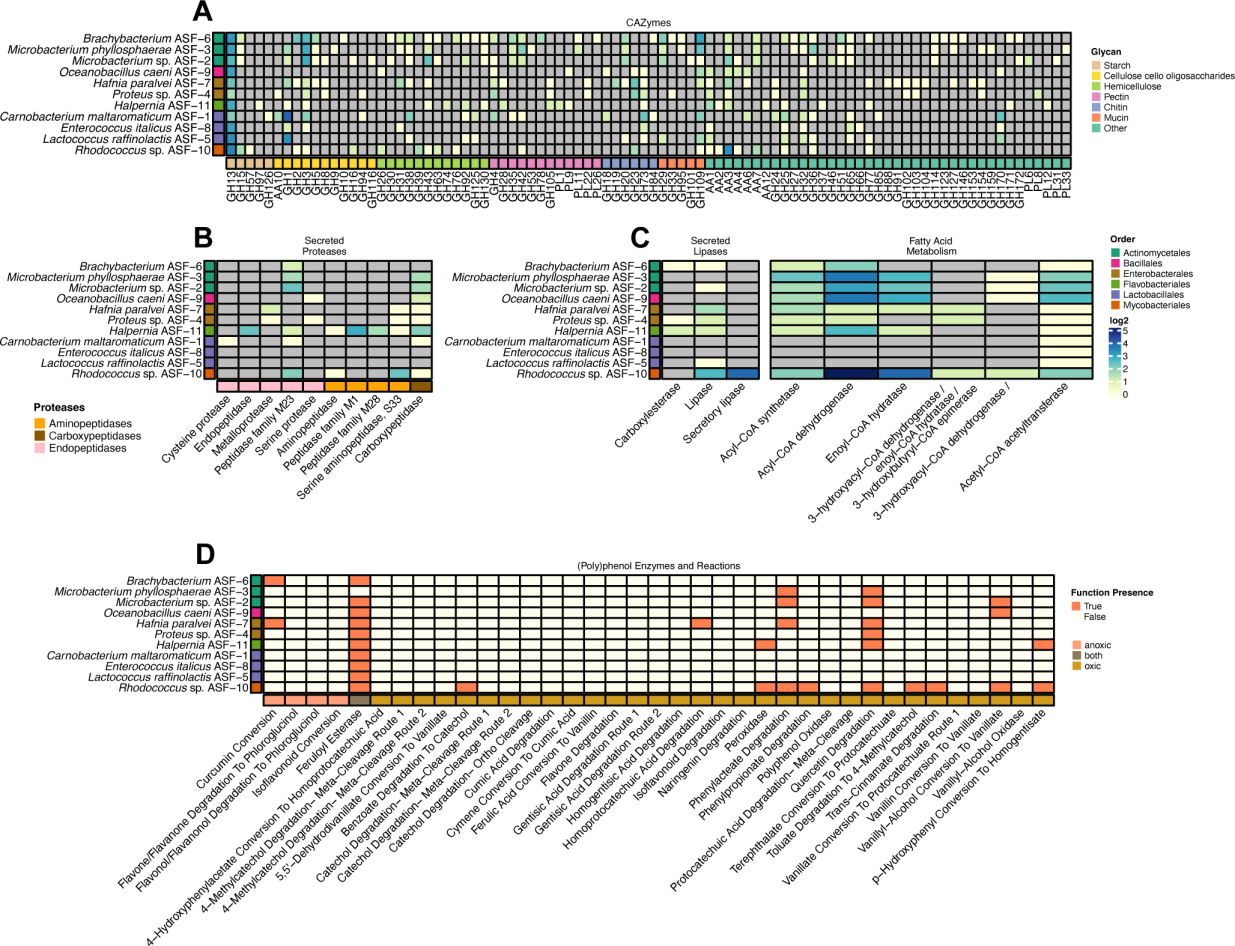
Metabolic potential for utilization of carbohydrates, proteins, lipids, and polyphenols from diet. (**A**) Heatmap illustrating the distribution of CAZymes (shown on x-axis) for each bacterial genome (y-axis). CAZymes are organized based on the different glycan substrates they target (1). (**B**) Heatmap illustrating the distribution of secreted proteases (shown on the x-axis) for each bacterial genome (y-axis). Proteases are organized in functional categories. (**C**) Heatmap illustrating the distribution of secreted lipases (shown on the x-axis) and enzymes involved in β-oxidation of fatty acids for each bacterial genome (y-axis). (**D**) Heatmap illustrating the presence (orange box) or absence (yellow box) of enzyme or metabolic pathways for utilization of polyphenols and aromatics (shown on the x-axis) for each bacterial genome (y-axis). The presence of genes encoding CAZymes, secreted proteases, or lipases is denoted by colored boxes that are weighted for copy number. CAZyme families, protease, and lipase categories that are not detected are represented by a gray box. In all panels, colors along the y-axis indicate the taxonomic order of each isolate.

Evolving aquaculture practices call for exploring novel feed components as sustainable alternatives to traditional fish feed-based diets (43, 64). Understanding how salmon fry gut microbes metabolize marine, plant, and insect-derived carbohydrates could guide novel strategies to optimize nutrition and health. To determine the capacity of the 11 isolates to utilize carbohydrates sourced from the diet, we investigated CAZyme family repertoires, and specifically the presence of GHs, carbohydrate esterases, polysaccharide lyases (PL), and auxiliary activities (AA; Fig. 4A and Table S1D). CAZyme families and subfamilies were associated with the utilization of selected fibers and host-derived glycans, based on experimentally characterized homologs as listed in the “characterized” section of each CAZyme family on https://www.cazy.org/. Most of the genomes contained, on average, 81 genes encoding CAZymes (Fig. 4A and Table S1D). The genome with the largest number of CAZymes was *Rhodococcus* sp. ASF-10 (number of CAZymes = 127), which is in line with previous studies in members of the same genus (65, 66), and suggests a possible substantial contribution of this bacterium to the metabolism of dietary components in the salmon gut. The dominant CAZyme family within the 11 bacteria was GH13 (Fig. 4A and Table S1D), which has been proven to have the capacity to metabolize starch (67), followed by GH1 and GH3 for manno-, xylo-, and cello-oligosaccharides depolymerization (Fig. 4A). CAZymes for depolymerization of hemicelluloses (xylan, GH10; mannan, GH26; arabinan, GH43 and GH51; manno-, xylo-, and cello-oligosaccharides, including GH2, GH3, GH9, and GH130), chitin (GH18, GH19, and GH20), mucin (GH95), and algal polysaccharides (GH16 and PL6 for agarose, laminarin, and alginate) were also detected.

#### Potential for utilization of dietary lipids, proteins, and their derivatives

Given the lipid- and protein-rich nature of fish diets, we investigated bacterial enzymes capable of processing these key nutrients (Fig. 4B and C and Table S1E and F). The genomes of nine bacteria, with the exception of *Enterococcus italicus* ASF-8 and *L. raffinolactis* ASF-5, harbor genes encoding secreted proteases and exopeptidases for degradation of proteins and short peptide chains, suggesting some levels of microbial protein breakdown in the gut environment (Fig. 4B and Table S1E). Genes encoding potentially secreted lipases and esterases were detected in seven genomes, with the exception of M. *phyllosphaera*e ASF-3, *C. maltaromaticum* ASF-1, *O. caeni* ASF-9, and *E. italicus* ASF-8, suggesting that a majority of the 11 bacterial isolates have a potential role in the utilization of feed-derived lipids and fatty acids (Fig. 4C and Table S1F). All bacteria except *C. maltaromaticum* ASF-1, *E. italicus* ASF-8, and *L. raffinolactis* ASF-5 could carry out medium and SCFA breakdown via the β-oxidation pathway to produce acetyl-CoA. This is consistent with previous studies showing that Lactobacillales lack the capacity to metabolize exogenous lipids and rely on them only for membrane synthesis (68). Overall, these findings suggest that several gut bacterial isolates possess enzymatic activities that may contribute to the digestion of lipids, proteins, and their short-chain derivatives, in line with the idea that they can either enhance nutrient availability in salmon fry or provide beneficial compounds resulting from their metabolic activity (69).

#### Potential for utilization of polyphenols and aromatics

Dietary polyphenols and flavonoids are natural compounds occurring in plants (70). Chemically, they are a heterogeneous group of hydroxylated phenyl compounds and are generally classified as flavonoids or nonflavonoids based on their structure and complexity. Research has shown that dietary polyphenols can modulate the composition of gut microbial communities in humans, and in turn, gut microbes are able to catabolize polyphenols and release bioactive metabolites (71). These metabolites have been associated with health-promoting effects, including their antioxidant, anti-inflammatory, and antibacterial properties. While plant phenolic compounds can be present in fish diets (such as daidzein from soybean) and have been frequently investigated as feed additives (such as quercetin, gallic acid, or rutin) to improve the growth and well-being of fish reared in RAS (72, 73), microbial polyphenol metabolism has never been explored in salmon gut microbiome studies. To identify potential transformation pathways of polymeric, monomeric, and simple phenols in the 11 bacterial isolates, we annotated their genomes using CAMPER (Fig. 4D and Table S1G). All isolates, with the exception of *M. phyllosphaera*e ASF-3, had genes coding for feruloyl esterases, enzymes that act on feruloylated polysaccharides such as arabinoxylans and pectins (74). *Rhodococcus* sp. ASF-10 showed the broadest ability to metabolize polyphenols, including pathways for degradation of flavonoids (quercetin), aromatic compounds related to polyphenol metabolism (phenylacetate, phenylpropionate, trans-cinnamate, and p-hydroxyphenyl), and vanillin derivatives. The two *Microbacterium* spp. showed potential for degradation of phenylacetate and quercetin, while enzymes for quercetin degradation were also observed in the genomes of *H. paralvei* ASF-7, *Halpernia* ASF-11, and *Proteus* sp. ASF-4. *Halpernia* ASF-11 showed potential for p-hydroxyphenyl conversion to homogentisate. Instead, *C. maltaromaticum* ASF-1 and *E. italicus* ASF-8 showed no potential for polyphenol utilization. Collectively, our data reveal a picture of potential microbiome–polyphenol interactions in the gut of salmon fry, highlighting the role of polyphenols, flavonoids, and vanillin derivatives as substrates that may influence microbial activity and host metabolism, despite being historically overlooked.

### Predicted capacity for vitamin production

**Fig 5:**
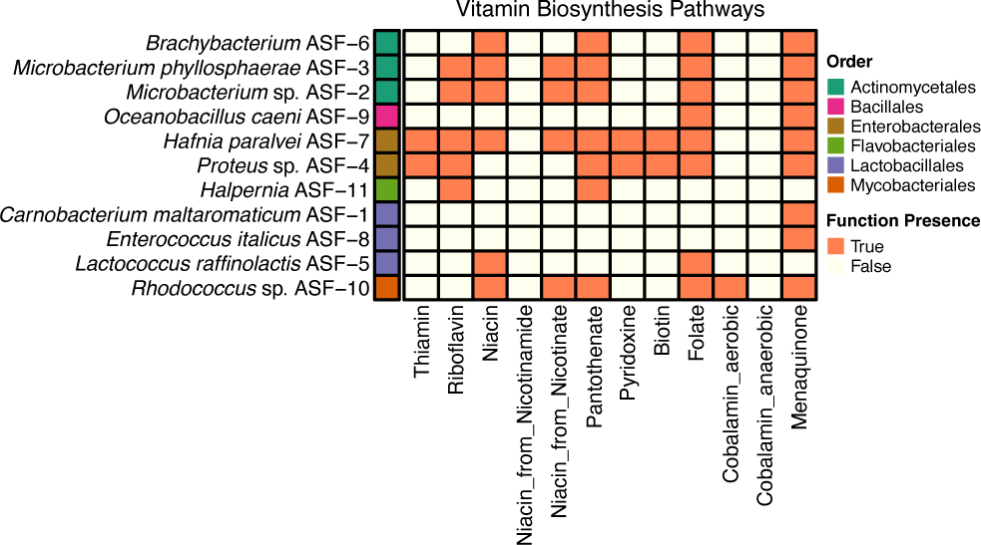
Heatmap showing the potential for vitamin biosynthesis pathways (x-axis) in the 11 bacterial isolates from the gut of Atlantic salmon fry (y-axis). Colors along the y-axis indicate the taxonomic order of each isolate. Orange boxes denote potential vitamin production, defined as the presence of at least 75% of the enzymes in the corresponding vitamin biosynthetic pathway.

B and K2 vitamins are micronutrients that serve as precursors for essential cofactors involved in diverse metabolic and regulatory processes, making them essential for both the host and its resident gut microbiota (75). Wild salmonids are highly sensitive to thiamine (B1) deficiency, which has been implicated in population declines across the Northern Hemisphere (76). In farmed fish, deficiencies in other vitamins such as riboflavin, niacin, pyridoxine, cobalamin, folate, and vitamin K can impair growth, development, and overall health. In addition, research has shown that salmon needs higher levels of B-vitamin when fed novel diets that are rich in plant-based ingredients (77). Although the gut microbiota is known to supply vitamin K and B-group vitamins to their hosts, as shown in humans and ruminants, their potential contribution to vitamin provision in salmon has received little, if any, attention. Given the high cost of supplements, strategies to enhance the vitamin biosynthetic capacity of the gut microbiota and reduce the need for exogenous vitamin supply are essential to lowering aquaculture production costs. To assess the vitamin biosynthetic potential of the 11 bacterial isolates in this study, we developed an in-house script encompassing 121 enzymes (EC numbers) involved in vitamin B and K biosynthesis, compiled through a comprehensive literature survey (Table S1H). K and B-vitamin biosynthetic potential was detected in all 11 isolates (Fig. 5). Specifically, *H. paralvei* ASF-7 and *Proteus* sp. ASF-4 have the genomic potential to produce thiamine. Riboflavin could be synthesized by *Microbacterium* sp. ASF-2, *M. phyllosphaerae* ASF-3, *H. paralvei* ASF-7, *Proteus* sp. ASF-4, and *Halpernia* ASF-11. *Brachybacterium* ASF-6, *Microbacterium* sp. ASF-2, *M. phyllosphaerae* ASF-3, *H. paralvei* ASF-7, *L. raffinolactis* ASF-5, and *Rhodococcus* sp. ASF-10 can produce niacin, while pyridoxine and biotin production seem to be exclusive abilities of *H. paralvei* ASF-7 and *Proteus* sp. ASF-4. Pantothenate, folate, and menaquinone (vitamin K) biosynthetic pathways were present in the genome of *Brachybacterium* ASF-6, *Microbacterium* sp. ASF-2, *M. phyllosphaerae* ASF-3, *H. paralvei* ASF-7, and *Rhodococcus* sp. ASF-10. Cobalamin biosynthetic potential was detected exclusively in the genome of *Rhodococcus* sp. ASF-10. Finally, *O. caeni* ASF-9 exhibited metabolic potential for the biosynthesis of folate and menaquinone only. Our results showed that microbial species with complementary vitamin biosynthetic potential are present in the gut of salmon fry, suggesting that interactions may involve the exchange of vitamins, their intermediates, and related metabolites across the microbial community. In addition, when released extracellularly, possibly through cell lysis, there could be a potential supply of vitamins to the salmon host, as previously postulated for Salmonid-related *Mycoplasmas* (78).

### Predicted capacity for secondary metabolite production

Secondary metabolites, such as bacteriocins, polyketides, non-ribosomal peptides, terpenes, siderophores, or antibiotics, are bioactive compounds that mediate competition and cooperation among microbes in the gut (79). In particular, they enhance niche competitiveness and suppress intestinal pathogens, a hallmark probiotic trait. We employed antiSMASH and BAGEL4 to identify putative secondary metabolite biosynthesis gene clusters in our 11 bacterial genomes (Table S1I and J). For bacteriocins (Table S1I), genes encoding sactipeptides were identified in the genomes of *Rhodococcus* ASF-10 and *C. maltaromaticum* ASF-1; the bacteriocin Zoocin_A in *E. italicus* ASF-8, while production of Colicin_E6, Alveicin_B_Bacteriocintoxin, and Bottromycin was predicted in *Proteus* sp. ASF-4; Lanthipeptide_class_II and Carnobacterium_MB1 in *C. maltaromaticum* ASF-1; as well as Lactococcin_972 in *L. raffinolactis* ASF-5. Several gene clusters for different secondary metabolite biosynthesis were detected (Table S1J). Terpene-precursor and type 3 polyketide synthase were the most widely distributed gene clusters, with almost all genomes having at least one of them. Clusters for the production of betalactone and RiPP-like (ribosomally synthesized and post-translationally modified peptide) compounds having antimicrobial or quorum-sensing roles (80) were detected in *M. phyllosphaerae* ASF-3, *H. paralvei* ASF-7, *Proteus* sp. ASF-4, *E. italicus* ASF-8, and *Rhodococcus* sp. ASF-10. The ectoine biosynthetic cluster (81) was identified in *Brachybacterium* ASF-6, *O. caeni* ASF-9, and *Rhodococcus* sp. ASF-10, suggesting that these isolates possess mechanisms for osmoprotection and are adapted to high-salinity environments. Similarly, the presence of Ni-siderophore clusters, previously shown to be involved in metal acquisition capabilities under iron-limited conditions (82), suggests that *Microbacterium* sp. ASF-2 and *H. paralvei* ASF-7 have this capacity. The detection of genes encoding bottromycin, an RiPP antibiotic (83) in *Brachybacterium* ASF-6, highlights its potential for producing this uncommon metabolite. Among all isolates, *Rhodococcus* sp. ASF-10 exhibits the most diverse secondary metabolite profile, encompassing gene clusters non-ribosomal peptide synthetase, type 1 polyketide synthase, terpene, and betalactone, underscoring its remarkable biosynthetic capacity and ecological versatility.

### Prediction of antibiotic resistance genes and prophage sequences

Assessing genomic safety features is a critical step in characterizing potentially beneficial bacterial strains. Our analysis indicates that 7 out of 11 bacteria isolated from salmon fry lack putative resistance genes, while *Microbacterium* sp. ASF-2, *M. phyllosphaerae* ASF-3, *H. paralvei* ASF-7, and *Proteus* sp. ASF-4 harbor genes for predicted resistance to tetracyclines [tet(42) and tet(H)] and β-lactam antibiotics (blaACC-1a) in their genomes (Table S1K). In addition, the genome of *Proteus* sp. ASF-4 includes the virulence gene *hugA* that encodes a heme/hemoglobin receptor protein known to help bacteria acquire iron from host heme or hemoglobin (84). The genomes of *Proteus* sp. ASF-4, *C. maltaromaticu*m ASF-1, *Microbacterium* sp. ASF-2, *H. paralvei* ASF-7, *O. caeni* ASF-9, *E. italicus* ASF-8, *Brachybacterium* ASF-6, and *L. raffinolactis* ASF-5 were found to contain prophage sequences (Table S2) of complete, high, and medium quality, as determined by CheckV (Table S2A). We identified transposase elements and gene coding for toxins such as YoeB in one of the prophages from *E. italicus* ASF−8 (Table S2B and C), consistent with previous literature (85). Intriguingly, *L. raffinolactis* ASF-5 harbors a predicted prophage with genes encoding proteins that could contribute to central carbon metabolism, redox balance, cell wall, and fatty acids synthesis (Table S2D). We did not identify any antibiotic resistance gene associated with prophage sequences.

### Evaluation of *L. raffinolactis* ASF-5 as a potential strategy to remove indigestible raffinose oligosaccharides from salmon feed

LAB, which produce lactic acid as a primary fermentation product from sugars, are generally regarded as beneficial due to their ability to antagonize bacterial pathogens and improve growth performance in aquatic animals (69, 86). Among them, the application of *Lactobacillus* spp. and *Lactococcus* spp. as probiotics in fish aquaculture has been extensively studied (87). However, while LAB are often identified as components of the gut microbiota of salmon, no indigenous species, adapted to the host and therefore with enhanced ability to establish in the gut, has been investigated as a potential beneficial bacterium for applications in salmon aquaculture. Equipped with our bacterial collection, we subsequently assessed the genetic and metabolic capacities of *L. raffinolactis* ASF-5 that could potentially contribute to host-beneficial effects.

**Fig 6:**
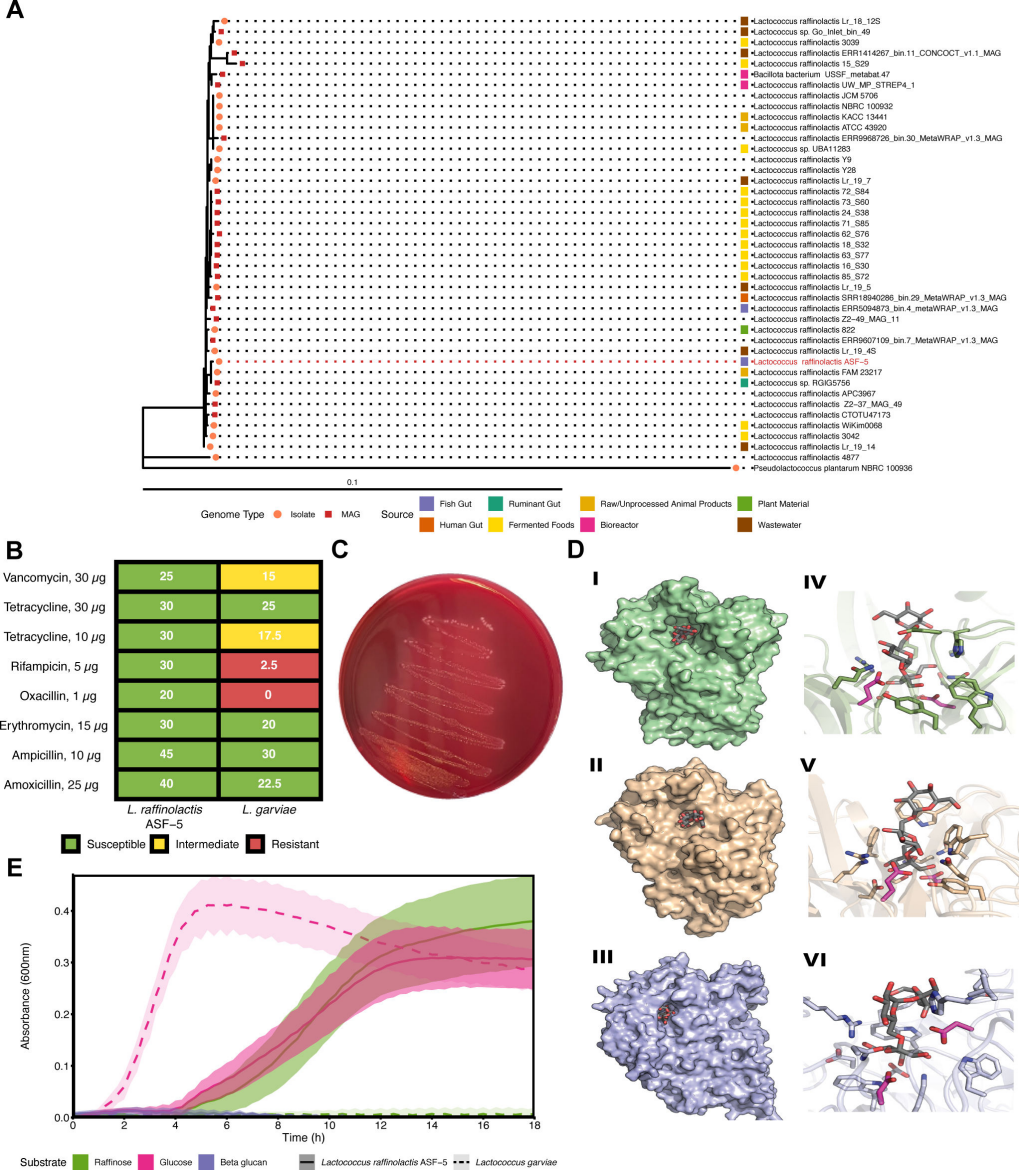
Phylogenomic analysis and functional characterization of *L. raffinolactis* ASF-5. (**A**) Phylogenetic tree showing the evolutionary relationships between *L. raffinolactis* ASF-5 and 42 publicly available *L. raffinolactis* genomes, including the outgroup *P. plantarum* NBRC 100936 (GCF_001591745.1). Genomes of isolates and metagenome-assembled genomes (MAGs) are marked by orange circles and red squares, respectively. The source of each strain is represented by the color of the squares next to the tip names. (**B**) Antimicrobial susceptibility of *L. raffinolactis* ASF-5 based on disc diffusion assays. The table shows halo diameters (millimeters) for each antimicrobial tested, with *L. garviae used* as a control in this assay. (**C**) Blood agar plate of *L. raffinolactis* ASF-5 showing no hemolytic activity indicated by the absence of a clearing zone. (**D**) Predicted enzyme–substrate complexes of the *L. raffinolactis*-derived GH32s and the GH36 with raffinose. Subpanels I–III show surface representations of the modeled complexes of LrGH32_1 (green), LrGH32_2 (tan), and LrGH36 (blue), each with the raffinose substrate bound in the active-site cavity as gray sticks. In all three enzymes, raffinose is buried within the catalytic cleft, consistent with substrate sequestration during hydrolysis. Panels IV–VI display close-up views of the active sites for LrGH32_1, LrGH32_2, and LrGH36, respectively, highlighting side chains within 4 Å of raffinose (colored as in subpanels I–III). Catalytic residues are shown in magenta sticks, while other interacting residues are depicted in their respective protein colors. The predicted substrate orientation and residue contacts were derived from the top-ranked AlphaFold 3 ligand pose for each enzyme. (**E**) Growth curves (OD₆₀₀) of *L. raffinolactis* ASF-5 and *L. garvieae* grown in MRS base supplemented with a single carbon source (raffinose, glucose, or β-glucan). Standard deviations from triplicates are shown as color-coded shaded areas, with substrates indicated by colors in the legend.

Despite the prevalence of this bacterium in dairy food products and its status of a species with demonstrated safety in the inventory of microbial food cultures (87, 88), and while often reported in fish gut ecosystems (69), little is known about the metabolic functions of this species in gut environments. Together with 42 genomic sequences representative of *L. raffinolactis* type strains and isolates from different sources, we reconstructed a phylogenomic tree based on 120 single-copy genes and determined relatedness and differences in genome size (Fig. 6A and Table S3). The closest related strains to *L. raffinolactis* ASF-5 were found to be *L. raffinolactis* FAM 23217 and *Lactococcus* sp. RGIG5756, isolated from rumen gut and raw animal products, respectively. Genome size was 2.05 Mb on average (1.32 Mb for the smallest and 2.63 Mb for the largest genome), which is consistent with the fact that *L. raffinolactis*, like other lactococci, has small genomes (89). Their relatively compact genomes reflect a highly specialized lifestyle to various environmental survival niches.

An important consideration in the initial selection of a strain to be used as a feed supplement is the assessment of its antibiotic resistance patterns. Although no antibiotic resistance genes were detected in the genomes of *L. raffinolactis* ASF-5, we further validated its susceptibility to antibiotics that inhibit cell wall synthesis, protein synthesis, and RNA synthesis, all relevant in aquaculture settings (90, 91), using the disc diffusion method (Fig. 6B). As a control, we used *L. garvieae*, a pathogen repeatedly isolated from animals including cattle and various fish species, which can cause lactococcosis in salmon (60). *L. raffinolactis* ASF-5 showed susceptibility to all tested antibiotics, while *L. garvieae* showed resistance to rifampicin and oxacillin, as previously reported (92, 93) as well as intermediate susceptibility to tetracycline and vancomycin. As an additional safety measure, probiotics must not exhibit hemolytic activity. We validate the absence of virulence factors such as hemolysin and cytolysin from the genomic prediction using a blood agar assay. Indeed, *L. raffinolactis* ASF-5 did not produce visible changes on blood agar both in either aerobic or anaerobic conditions (Fig. 6C).

Unlike most lactococci, *L. raffinolactis* can ferment α-galactosides, including melibiose and raffinose (94). The raffinose family oligosaccharides (RFOs) are ubiquitous in legume seeds and common in human and animal diets. While removal of RFOs from pulse-based ingredients (such as soybean meals) allows these products to be consumed without digestive problems, emerging research highlights the potential prebiotic effects of raffinose, as it can influence gut microbial composition by boosting the growth of beneficial bacteria, including LAB (95). In the search for novel plant-based protein ingredients in aquafeed, raffinose is present in peas and fava bean-derived meals, from crops that are particularly relevant in boreal climates (96). Pre-fermentation of the feed or coadministration with a bacterium possessing enzymatic capabilities for the utilization of this sugar could reduce the undesirable physiological effects associated with raffinose consumption while enhancing its beneficial effects for the host. In addition, enzymes derived from this bacterium for raffinose processing could be used as a cell-free strategy and supplemented to feed and increase its digestibility.

In the *L. raffinolactis* ASF-5 genome, the presence of genes encoding a GH36 α-galactosidase (LrGH36, contig_1_594) and two GH32 (LrGH32_1, contig_1_466; LrGH32_2, contig_2_39) for hydrolysis of sucrose into glucose and fructose suggests a capacity for complete depolymerization of raffinose. We generated high-confidence 3D structural models of the two LrGH32 and LrGH36 and modeled enzyme-substrate interactions through docking using AlphaFold3. The predicted complexes showed raffinose bound deeply within the catalytic cleft of the enzymes (Fig. 6D), stabilized by residues consistent with known GH32 and GH36 (97). These results support that LrGH32 and LrGH36 likely cooperate in the complete hydrolysis of raffinose, enabling *L. raffinolactis* ASF-5 to utilize this trisaccharide as a carbon source.

To support the genome-based functional inferences, we further tested the ability of *L. raffinolactis* ASF-5 to grow on raffinose. The results of the growth experiments indicated that *L. raffinolactis* could indeed grow on raffinose, while the same carbohydrate did not support the growth of the pathogenic *L. garvieae* (Fig. 6E).

Overall, genotypic and phenotypic assays show that *L. raffinolactis* ASF-5 fulfills safety criteria and can produce metabolites such as bacteriocins (Table S1I), L-lactate (Fig. 3), vitamin B9 (folate; Fig. 5) that contribute to higher niche competitiveness, inhibition of intestinal pathogens, and provision of vitamins to the host. This suggests that coadministration of this beneficial bacterium, or addition of recombinantly produced and purified LrGH32s and LrGH36, to feeds containing raffinose could selectively promote the growth of *L. raffinolactis* ASF-5, remove detrimental components for salmon digestion, and potentially improve the health and welfare of farmed Atlantic salmon. As a next step, further research is required to assess the applicability of this strain in fish trials.

### Conclusions

In this study, we isolated and genotypically characterized a selection of bacteria from the GIT of salmon fry. Genome screening revealed a wide array of CAZymes, proteases, lipases, polyphenol, and aromatics-degrading enzymes, reflecting the metabolic diversity of these organisms and their potential to contribute to the metabolism of dietary components. We identified several beneficial properties, expanding the understanding of individual vitamin B and K production by salmon gut commensals, as well as bacteriocins and other secondary metabolites that could improve niche competitiveness and suppress intestinal pathogens. Genotypic and phenotypic screening, as well as assessment of safety properties, revealed that *L. raffinolactis* ASF-5, or its GH32s and GH36 enzymes, has the potential to be used for removing indigestible components, such as raffinose, in salmon feeds. Overall, our findings represent a step forward in uncovering how gut bacteria in early stages of the fish life may interact with dietary components and their functions that can contribute to salmon health and nutrition. This is particularly relevant as it can open avenues for developing microbe-based or microbiota-based interventions tailored to farmed salmon, with the aim of influencing and optimizing microbial communities’ composition and functions that support health and resilience in aquaculture systems. Of note, bacterial isolates and their enzymes can also be exploited for biotechnological applications aimed at improving nutrient digestibility in plant-based feeds. Additional *in vivo* investigations are needed to fully evaluate this potential.

## Data Availability

Oxford Nanopore sequencing reads have been deposited in the SRA with project number PRJEB103841. The genomes and their corresponding annotations are publicly available via Figshare (https://doi.org/10.6084/m9.figshare.30731261).

## SUPPLEMENTAL MATERIAL

Table S1 aem.02409-25-s0001.xlsx Overview of the source, genome size, completeness, contamination, and taxonomy, as well as detected genes encoding CAZymes, enzymes for metabolism of polyphenols and aromatics, proteolytic enzymes, lipases, enzymes for fatty acid metabolism, bacteriocins, secondary metabolites, and antibiotic resistance genes detected in the genomes of the 11 bacterial isolates presented in this study.

Table S2 aem.02409-25-s0002.xlsx Predicted DNA and RNA viral signatures in the genomes of the 11 bacterial isolates.

Table S3 aem.02409-25-s0003.xlsx Summary of metadata used to construct the phylogenomic tree presented in Fig. 6A.

ASM does not own the copyrights to Supplemental Material that may be linked to, or accessed through, an article. The authors have granted ASM a non-exclusive, world-wide license to publish the Supplemental Material files. Please contact the corresponding author directly for reuse.
